# How the Human Brain Segments Continuous Experience

**DOI:** 10.1523/JNEUROSCI.3041-18.2019

**Published:** 2019-04-24

**Authors:** Angharad N. Williams, Mark Postans, Carl J. Hodgetts

**Affiliations:** Cardiff University Brain Research Imaging Centre (CUBRIC), School of Psychology, Cardiff University, Cardiff CF10 3AT, United Kingdom

The formation and subsequent recall of coherent episodes from our past (i.e., our episodic memories) relies on the ability to parse the continuous stream of experience in our daily lives into meaningful chunks or segments (Zacks et al., 2010). This segmentation is thought to determine how episodic memories are organized. For instance, elements within an event are bound together more so than individual elements across events (DuBrow and Davachi, 2013, 2016), and the ability to segment information is linked to better subsequent memory (Sargent et al., 2013).

The events we experience and remember are often complex and unfold over time, but neuroimaging studies of episodic memory have typically used isolated static stimuli. Despite providing tight control over what is seen, and for how long, there is clearly a disconnect between classic laboratory tests of episodic memory and the construction of event representations in everyday life. Therefore, researchers have sought to use stimuli that are more dynamic and naturalistic, such as discrete film-clips lasting a few seconds. Such studies have found that hippocampal activity increases at the immediate offset of clips and is greater for remembered versus forgotten episodes (Ben-Yakov and Dudai, 2011; Ben-Yakov et al., 2013). The authors suggested that this signal might reflect the laying down of integrated event memories. These studies, however, did not examine how the hippocampus responds during continuous experience.

To address this question, a recent study in *The Journal of Neuroscience* (Ben-Yakov and Henson, 2018) analyzed functional magnetic resonance imaging (fMRI) data collected as participants viewed an 8 min version of Alfred Hitchcock's drama “Bang! You're Dead,” or a longer feature-length version of the film Forrest Gump. To define event segments, or “boundaries,” an independent set of participants was asked to watch the two movies and press a button whenever they felt an event (“one meaningful unit”) ended and another began. These annotations were then used to examine hippocampal activity at event boundaries in the two fMRI datasets. Ben-Yakov and Henson (2018) noted a number of possible perceptual confounds (e.g., shifts in luminance, color histograms, or auditory similarity across the frames of each boundary), but took steps to control for these in their analyses. It is important to note that the way movies are created and edited leads to potentially dramatic changes in visual and/or auditory factors. For example, films are composed of a series of scenes with abrupt changes in location, characters, and other elements. Therefore, although they are more similar to real life than the static elements used in previous experiments, some of the boundaries are unlike the continuous transition between events in real life.

Going beyond previous work linking the hippocampus to event-boundary processing (Ben-Yakov and Dudai, 2011; Ben-Yakov et al., 2013; DuBrow and Davachi, 2016), Ben-Yakov and Henson (2018) demonstrated that hippocampal activity increased at the event boundaries, and that activity was greater for boundaries for which there was high agreement between independent raters. The latter effect remained significant for the longer movie even when controlling for all of the identified perceptual confounds. The authors noted that the shorter movie possibly contained too few boundaries relative to the total number of confounds, though the effect remained for both movies when each covariate was added to the model separately (Ben-Yakov and Henson, 2018). Notably, Zacks et al. (2010) previously found that perceptual changes (in the physical stimulus, e.g., color/location) were not always sufficient to establish a neural response at event boundaries. Some responses, for example, corresponded with more conceptual changes, involving interactions between characters, their goals and intentions, thus underlining the range of properties that shape episodic context, over and above perceptual factors (Brunec et al., 2018).

This finding of Ben-Yakov and Henson (2018) dovetails with that of a previous study (Baldassano et al., 2017) that applied a computational approach to identify temporal structure in neuroimaging data. Baldassano et al. (2017) found that during continuous movie viewing, shifts in cortical activity patterns (including in angular gyrus, posterior medial cortex, and parahippocampal cortex) coincided with increased hippocampal activity. In addition, the cortical activity patterns matched annotated event boundaries 35–40% of the time. Building on this, Ben-Yakov and Henson (2018) sought to directly test the relationship between event boundaries and hippocampal activity. Here, they used a similar method to detect peaks in hippocampal activity across time, and subsequently examined the alignment between these peaks and event boundaries. Indeed, a significant correspondence was found between the strongest hippocampal responses and the annotated event boundaries. There were, however, additional peaks in hippocampal activity that did not correspond with the independently defined boundaries.

In this context, a potential limitation of this work is that the segmentations were determined by an independent sample of participants who did not undergo brain imaging. Critically, the information that defines an event boundary, and the granularity at which they are placed, may vary across individuals according to what feels natural to them. Ben-Yakov and Henson (2018) also conducted a binned salience analysis (low/medium/high) according to the number of observers identifying a boundary at a similar point in time. However, despite demonstrating that hippocampal activity was modulated by their coarse “boundary salience” measure, the authors appropriately concluded that it could reflect a measure of boundary strength, coarser levels of event segmentation, or the likelihood of agreement across observers. A question for future work, therefore, is how the brain simultaneously processes events of varying temporal (and spatial) scales, and how this may differ between individuals (Brunec et al., 2018). Sargent et al. (2013) found that segmentation ability (defined as the degree to which an individual agreed with the sample) was related to better memory performance (the number of character actions correctly recalled after movie viewing). Thus, interindividual differences in segmentation ability may account for individual variation in event memory.

Different types of information or types of boundaries may be represented by distinct hippocampal networks (Ranganath and Ritchey, 2012; Robin and Moscovitch, 2017; Murray et al., 2018). For example, the processing of spatial, temporal, perceptual, and conceptual boundaries may involve hippocampal interactions with different cortical and subcortical areas (Brunec et al., 2018). Moreover, the hippocampus itself is a highly heterogeneous structure, comprising multiple subfields (Berron et al., 2017; Hodgetts et al., 2017; Dimsdale-Zucker et al., 2018) and subdivisions along its anterior–posterior axis that have distinct connectivity profiles (Strange et al., 2014; Aggleton et al., 2015). Therefore, different hippocampal subregions might play distinct but complementary roles in event segmentation and memory depending on their connectivity with surrounding areas.

Subregions of the prefrontal cortex (PFC) may also support different aspects of event memory (Clewett and Davachi, 2017). Specifically, functional connectivity between the hippocampus and medial PFC has been linked to memory integration within an event, whereas activity in both lateral PFC and the hippocampus at boundaries has been related to the ability to associate memories across discrete events (DuBrow and Davachi, 2016). Based on these results, Clewett and Davachi (2017) suggested that hippocampal retrieval mechanisms, through dynamic interactions with the PFC, shape the memory integration processes that operate during encoding. They proposed that hippocampal retrieval processes may prioritize reactivation of the just-encoded information for the purpose of integration. Consistent with this, a related electroencephalography study demonstrated that the rapid replay of preceding events, triggered by boundaries, predicted subsequent sequential recall over those boundaries (Sols et al., 2017).

A key unanswered question is what these increases in hippocampal activity at event boundaries represent. As suggested above, they may be interpreted as a rapid replay of the just encountered event to create a cohesive representation (Ben-Yakov and Dudai, 2011). Alternatively, this hippocampal signal may reflect (or even trigger) a rapid shift in situation models (mental representations that summarize the temporal and contextual details of an event), which in themselves may be represented within an extended posterior hippocampal network (Ranganath and Ritchey, 2012; Baldassano et al., 2017; DuBrow et al., 2017). Further studies incorporating both event segmentation and memory will be required to adjudicate between these possible explanations.

In summary, Ben-Yakov and Henson (2018) provide compelling evidence for the role of the hippocampus in segmenting our continuous experience. They find that peaks in hippocampal activity are both sensitive to and specific to subjective event boundaries during naturalistic experience, as assessed using movie viewing. These findings highlight the utility of complex, naturalistic stimuli for the study of hippocampal contributions to event segmentation and memory, but they also illustrate the unique theoretical and methodological challenges that arise as we move toward more unconstrained, ecologically valid paradigms in cognitive psychology and fMRI.
